# Measuring domestic violence against Egyptian women and its consequent cost using a latent variable model

**DOI:** 10.1186/s12905-024-03465-6

**Published:** 2024-12-02

**Authors:** Mai Sherif Hafez, Carolin Sherif Mounir, Laila Othman El Zeini

**Affiliations:** Department of Statistics, Faculty of Economics and Political Science, Cairo University, Cairo, Egypt

**Keywords:** Domestic violence, Cost of violence, Latent variable models, Latent trait model

## Abstract

**Background:**

Domestic Violence is a threatening worldwide problem. Its consequences against women can be dramatic, as it negatively affects women’s quality of life reflected in their general wellbeing including physical, mental, emotional and sexual health, in addition to the economic cost. Both domestic violence and its cost are multidimensional constructs that cannot be directly measured.

**Methodology:**

In this study, a latent trait model is used by applying item response theory to measure both domestic violence and its consequent cost via thirty-five observed variables. Accordingly, the study fills a gap in the literature since it is the first attempt to examine the relationship between domestic violence and its consequent cost in Egypt using latent variable modelling rather than simple descriptive statistics. Each construct is considered as a multidimensional latent variable. The overall latent trait model also estimates the relationship between domestic violence and its consequent cost. The effect of a number of socioeconomic covariates on domestic violence is examined within the model. The proposed model is fitted to data from the 2015 Egypt Economic Cost of Gender-Based Violence Survey (ECGBVS) using Mplus software.

**Results:**

The study shows that psychological violence is equally important in measuring domestic violence, as physical violence. The cost resulting from domestic violence relies in its measurement both on the reduced quality of life and the monetary cost endured by the violated woman and children. For socioeconomic covariates, it is shown that domestic violence is affected by women’s and husband’s age, educational level, and husband’s occupational status.

**Conclusion:**

Domestic violence is measured by summarizing four forms of violence: physical, psychological, sexual and economic violence, in a single continuous latent variable measuring “Domestic Violence”. Similarly, Cost is measured by summarizing three forms of consequent cost of violence: economic cost, cost on children and cost on women’s quality of life, in another a single continuous latent variable “Cost”. Each of these dimensions is measured by a number of aspects, reflecting the multidimensional nature of the variables. The fitted latent trait model ensured the positive relationship between Domestic Violence and its consequent multidimensional cost.

**Supplementary Information:**

The online version contains supplementary material available at 10.1186/s12905-024-03465-6.

## Background

“Violence against women is perhaps the most shameful human rights violation. And it is perhaps the most pervasive. It knows no boundaries of geography, culture or wealth. As long as it continues, we cannot claim to be making real progress towards equality, development and peace.” (Former UN Secretary-General Kofi Annan).

Violence against women is considered as a serious social problem, a criminal offence and human rights violation of worldwide significance. It occurs in all countries, regardless of social, economic, religious, or cultural background. Global estimates of the World Health Organization [1] showed that almost 1 in 3 women (30%) had faced either physical or/and sexual violence by their intimate partners, known as intimate partner violence (IPV). They also show that male intimate partners commit 38% of murders of women.

The United Nations declaration on the elimination of violence against women adopted by the UN general Assembly [2] defines violence against women as “any act of gender-based violence that results in, or is likely to result in, physical, sexual, or mental harm or suffering to women, including threats of such acts, coercion or arbitrary deprivation of liberty, whether occurring in public or in private life.” The most common and widespread form of violence is domestic violence (DV) or intimate partner violence (IPV), which refers to “behaviour by an intimate partner or ex-partner that causes physical, sexual or psychological harm, including physical aggression, sexual coercion, psychological abuse and controlling behaviours.”

Governmental and non-governmental organizations around the world have long targeted and raised awareness towards violence against women as a worldwide increasing threaten as well as an important public health problem. Special attention is given to domestic violence, which acts as an important risk factor to women’s health, on physical, psychological, and mental levels. In its resolution, the UN general assembly [3] reported Sustainable Development Goals (SDGs) that countries are committed to implement by 2030. The fifth goal focuses on achieving gender equality and women empowerment, targeting to eliminate all forms of violence against women and girls in the public and private spheres, including trafficking and sexual, and other types of exploitation.

Consequences of domestic violence can be severe, affecting woman’s health by causing injuries, burns and sexual problems like reproductive issues, or sexually transmitted diseases, including HIV/AIDS [4–6]. Violence may also have its effects on woman’s mental health as it causes anxiety, stress, sleeping difficulties, difficulties in daily activity, affecting her wellbeing, and possibly reaching suicidal attempts [7–10]. The cost of violence can be economic too, since violated women may spend money to get medical services needed to heal physically or mentally, in addition to fees for legal services, in case she decides to resort to justice. Children are quite vulnerable to domestic violence too, as children witnessing parental violence are more likely to suffer from mental disorders or low educational achievement, as reported in the literature [11, 12].

It is worth mentioning that due to Egyptian culture and traditions, partners do not usually cohabit outside the frame of marriage. Accordingly, the term “Spousal Violence” is often used in local studies, rather than “Intimate Partner Violence”. Thus, “Spousal Violence” or “Domestic Violence” will be used interchangeably throughout this study.

## Literature review

Research about violence against women can be found in fields of sociology, psychology, public health, and various other fields depending on the perspective of the study. Relevant studies that have focused on the measurement of Intimate Partner Violence in relation to other sociological phenomena, in different parts of the world, are outlined below.

Many researchers concerned with the measurement of Intimate Partner Violence opted to label a woman as physically, sexually, emotionally, or economically violated if she responds affirmatively to at least one of a list of questions related to the relevant type of violence. The measured variable can then be used within the chosen statistical model to study relationships with other explanatory variables or factors of interest.

Nwabunike & Tenkorang [13] studied domestic and spousal violence against women in Nigeria among three selected ethnic groups, using data from 2008 Nigeria Demographic and Health Survey. Physical violence was created from answers to six questions, while sexual violence was created from two questions and Emotional violence was created from four questions. Any type of violence variables were coded as “yes = 1” if a woman answered affirmatively to any of the related questions and coded as “no = 0” otherwise. The results of a complementary log-log model showed that ethnicity is significantly correlated with physical, sexual, and emotional violence. Women faced family violence during childhood were more likely to report physical and sexual violence.

Alkan et al. [14–17] conducted separate studies to detect factors that affect economic violence, sexual violence, verbal and psychological violence and controlling behaviour by husband against women in Turkey. Each of these studies used cross-sectional data of National research on domestic violence against women in Turkey, conducted by the Hacettepe University Institute of Population Studies in 2008 and 2014 [14, 17]. applied binary logistic regression analysis in order to determine factors affecting economic violence against women and women’s status of being exposed to controlling behaviour, respectively. While [15, 16] performed binary logistic regression and binary probit regression analyses to determine the factors affecting sexual violence and psychological violence, respectively. It was found that women’s exposure to various types of violence was affected by different socioeconomic factors including region, age, level of education, employment status, health condition, marital status, and number of children. Women with more children were more likely to face economic violence while less likely to expose to sexual violence compared to those with no children. The higher a woman’s educational level, the less likely to expose to sexual violence, and the more likely she is to be exposed to verbal, psychological and economic violence. Additionally, it was found that employed women were more likely to experience sexual violence and less likely to be exposed to economic violence compared to those who were unemployed. According to the findings, the risk of being exposed to controlling behaviour was found to be higher for women who are exposed to economic violence. Finally, women residing in urban areas were more likely to experience economic violence compared to those living in rural, but less likely to face sexual violence and controlling behaviour.

Other studies used different types of latent variable models to measure types of Domestic Violence, and to model its relationship with other factors.

Ribeiro et al. [18] aimed to assess the effects of social support and socioeconomic factors on violated pregnant Brazilian women using structural equation models. The study proposed three main latent variables: socioeconomic factor, social support factor and general violence factor. Socioeconomic latent variable was measured using four items: education of the pregnant woman, occupation of the family head, monthly family income in minimum wages and economic class. The general violence latent variable was measured using three latent variables: Physical violence, psychological violence, and sexual violence, which were measured using 13 questions about violence defined by the WHO. Social support was measured using four latent factors, proposed by the Medical Outcomes Study (MOS): tangible support (four questions), emotional support (seven questions), affectionate support (three questions), and positive social interaction support (four questions). Results showed that in general the higher the socioeconomic level, the higher the social support and lower exposure to violence.

Latent Class Analysis was used in a number of studies, to identify patterns of IPV in terms of severity (low/high) and type of violence (physical/sexual). In Mexico, Gupta et al. [19] conducted a study on low-income women to study different patterns of domestic violence and associations with work-related disruptions. Results of separate multilevel risk regression analyses showed that work disruption was highly related to physical IPV and the existence of injuries. As an extension for the previous study, Scolese & Gupta et al. [20] studied the association between patterns of IPV against low-income women in Mexico and child school attendance. Child schooling disruption was found to be highly related with physical IPV.

In Nepal, Clark et al. [21] used a Latent Class Analysis to distinguish different classes of IPV, and to study the relationship between IPV and women’s exhibiting symptoms of depression. Four classes of violence were detected indicating different levels of severity and type of violence (physical/sexual). Results of a multilevel negative binomial regression model showed that all classes with higher levels of violence were associated with more cases that exhibited symptoms of depression relative to the low violence class, adjusting for age, educational level, household financial stress, prior exposure to child maltreatment, self-efficacy, and natal and in-law family relationship quality.

In Egypt, the analysis of Domestic Violence is mostly limited to studies that use logistic regression, where the outcome variable is some binary variable that indicates whether a woman has been subject to violence, or not; in addition to exploratory factor analysis. Akmatov et al. [22] compared factors associated with wife beating in Egypt, using data from the 1995 and 2005 Egyptian Demographic and Health Surveys. Results show that prevalence of wife beating in 2005 was higher than in 1995, with a weaker association between socio-demographic differentials and wife beating in 2005. Factor analysis results uncovered three levels of violence: extreme, strong and moderate.

Yount [23] studied the effect of household wealth, women’s social and economic dependence on husband, work status, number of children, among other demographic factors on physical violence against Egyptian women in Minya. Results of logistic regressions showed that there was a strong negative relationship between household wealth and wife beating. Lower education levels, more number of children, younger ages of women were found to be positively related to wife beating. The study also showed that women’s working status, and living with parents in law, along with other demographic factors were uncorrelated with facing violence.

In an attempt to measure the economic cost of spousal violence against women in Egypt, the Central Agency for Public Mobilization and Statistics (CAPMAS) in cooperation with United Nations Population Fund (UNFPA) and the National Council for Women (NCW) conducted the Egypt Economic Cost of Gender- Based Violence Survey (ECGBVS, 2015) that allows measuring violence against women and its cost. ECGBVS measured three types of domestic violence: physical, psychological, and sexual. The survey’s report [24] considered a woman as physically abused by her husband if she responded affirmatively to at least one of the six questions related to the physical violence. The same is implemented to measure sexual and psychological violence. ECGBVS defined four kinds of cost that women could face as a result of being exposed to spousal violence: direct tangible cost, direct intangible cost, indirect tangible cost, and indirect intangible cost. The economic cost of violence was measured in the survey by materializing and monetarizing all aspects of cost.

Latent variable modelling of the dimensions of violence is clearly lacking in the studies about Egypt. Most studies classify women as either violated or not by considering a woman to be violated if she responds positively to at least one of a set of questions related to violence, thus discarding the severity and/or the type of violence, as well as ignoring weights of its determinants. Worldwide, few studies considered the cost of exposure to domestic violence, most of which focused on the economic dimension of cost. In Egypt, no previous studies addressed the consequent cost of facing domestic violence, expect for ECGBVS which focused merely on measuring the economic cost of facing violence, by converting the cost of any endured violence (e.g. injuries, work or school disruption, psychological problems,…etc.) in terms of money using simple calculations and assumptions, that do not involve modelling, and discard the multidimensionality of the phenomenon.

The contribution of this study lies within two main areas; the development of the measurement aspect of the latent constructs (violence and its consequent cost), and the structural modelling of the relationship between them. This study proposes a structural equation model that measures both domestic violence and its consequent cost as multidimensional latent variables, allowing for different dimensions of each construct to be measured and weighted according to their relevance and importance. The proposed model also attempts to examine the relationship between domestic violence and its cost using latent variable models. It is also a first attempt to further analyze the 2015 ECGBVS data, providing an in-depth understating for domestic violence against Egyptian women, its determinants, and its consequent cost, thus providing more information needed for decision making. The proposed model can be used on datasets from other countries to provide a study of the same phenomenon, or to compare with other societies.

## Methods

### Methodology

This study aims to measure domestic violence and its consequent cost, via a latent variable model. Both domestic violence and its consequent cost are multidimensional constructs that cannot be directly measured. Domestic violence is measured over two levels. The first level measures different aspects of violence including physical, sexual, psychological and economic violence, using 17 observed variables. The second level summarizes these aspects in one variable, giving different weights to the different dimensions of violence, using a latent trait model. Consequent cost of facing spousal violence is also measured over two levels. The first level measures different aspects of cost, going beyond the economic cost to include cost on quality of life and cost on children, based on 18 observed variables. The second level summarizes these aspects in one variable, using another latent trait model.

**Fig. 1:**
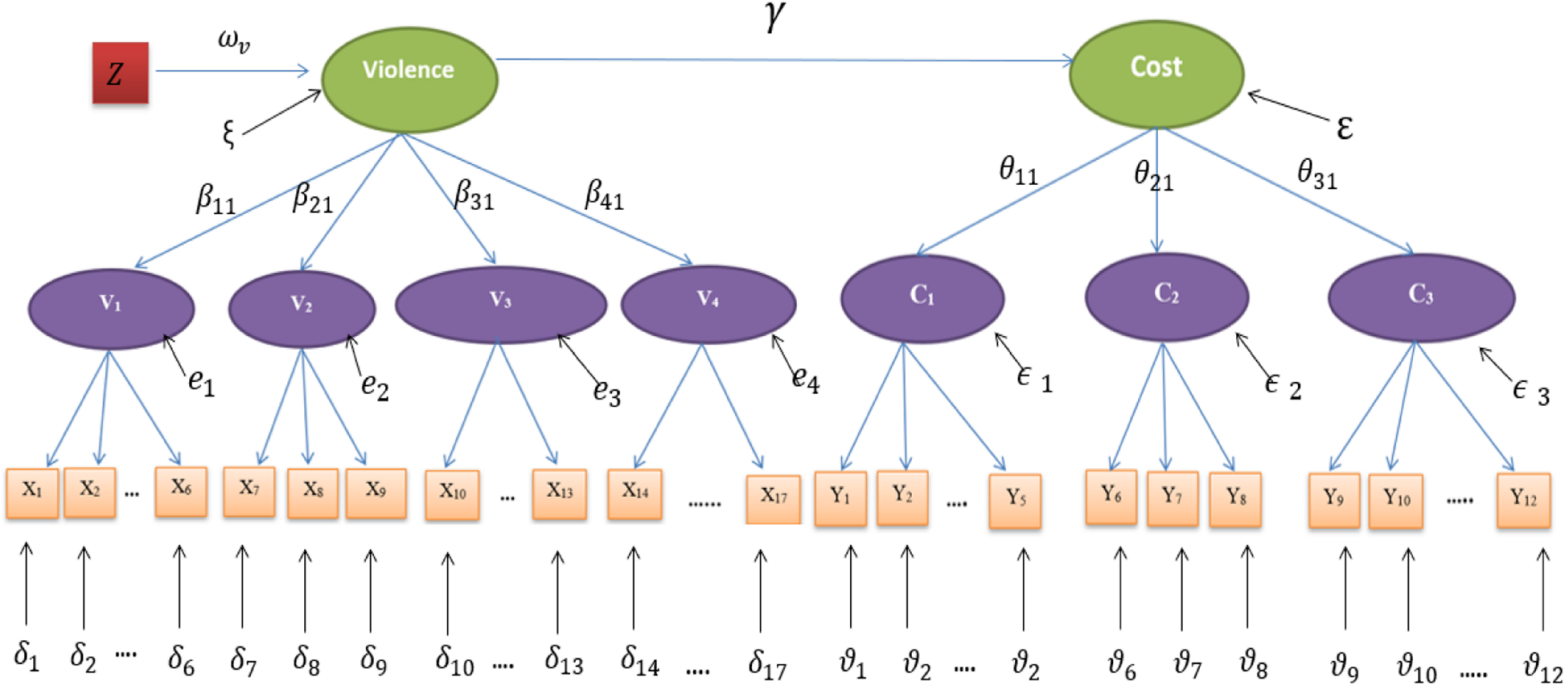
Path diagram representing a latent variable model for the relationship of domestic violence and its consequent cost

The model assumes that metric latent variables are used to explain the relations among categorical/binary observed variables. In case of binary manifest variables, the variables are recorded as 1 or 0; 1 indicating a positive response, that is “yes”, and 0 indicating a negative response [25, 26]. Item Response Theory (IRT) approach will be adopted in modelling both violence and its consequent cost using logit link functions. Structural Equation Models (SEM) are employed to study the relationship between violence and its cost within the full model, in addition to assessing the effect of socioeconomic covariates on violence. To provide an in-depth understanding of domestic violence against Egyptian women, its determinants, and its cost, the proposed model is fitted to data from the 2015 Egypt Economic Cost of Gender-Based Violence Survey (ECGBVS). Figure 1 represents a hypothesized path diagram for the proposed model.

The first part of this model is designed to measure domestic violence against women. This is carried out on two levels. The first level (the measurement model) measures the different aspects of violence, physical, sexual, psychological and economic violence that women may face (denoted by V_1_, V_2,_ V_3,_ V_4_ respectively in Fig. 1), as latent variables, using a latent trait model. The second level (the structural model) summarizes these different aspects of violence via a single continuous latent variable that we refer to as “Violence”. This model can be expressed by the following set of equations. The measurement model is represented by

1 $$\:\text{L}\text{o}\text{g}\text{i}\text{t}\:{\pi\:}_{ij}\left(\varvec{v}\right)\:=\:{{\alpha\:}_{i0}}^{\left(x\right)}+{\alpha\:}_{ij}{v}_{j}+{\delta\:}_{i},$$

where *i* = 1,2,…, *p*, represents the index for manifest variables and *j =* 1,2,*…*,*q* denotes the index for latent variables, *p* is the number of manifest variables and *q* denotes the number of latent variables.$\:\:{\pi\:}_{ij}\left(\varvec{v}\right)$ is the conditional probability that $\:{x}_{i}$ has a positive response given the values of the $\:q$ latent variables $\:{v}_{1},\dots\:\dots\:.,{v}_{q}$, while ***v*** represents a vector of *q* latent variables that measure the different aspects of violence. In the measurement model, $\:{{\alpha\:}_{i0}}^{\left(x\right)}\:and\:{\alpha\:}_{ij}$ represent intercept and factor loadings, respectively.

The structural part is presented as

2 $$\:{v}_{j}=\:{\beta\:}_{j1}\:violence+{e}_{j},\:j=\:\text{1,2},\:\dots\:.q$$

where $\:{\beta\:}_{j1}\:$represents the factor loadings of the overall latent variable “violence” over the various dimensions of violence.

The second part of the model is designed to measure the cost that women bear as a consequence of violence, following the same structure as that of violence. The measurement part will measure different aspects of cost such as, cost on health (physical, mental or monetary cost), cost on children who witness domestic violence, economic (monetary) cost that a woman may pay due to violence or cost on her wellbeing. These aspects are measured as latent variables (denoted by C_1_, C_2_, and C_3_, respectively) using a latent trait model, while the structural part summarizes these different aspects of cost via a single continuous latent variable that we refer to as “Cost”. The choice of the manifest variables and labelling of latent variables was implemented following an exploratory factor analysis, that suggested the use of three factors. This model can be expressed by the following set of equations. The measurement model is represented as

3 $$\:Logit\:{\pi\:}_{kl}\left(\varvec{c}\right)\:\:=\:{{\alpha\:}_{k0\left(s\right)}}^{\left(y\right)}+{\alpha\:}_{kl}{c}_{l}+{\vartheta\:}_{k}$$

where *k* = 1,2,…, *n*, represents the index for manifest variables and *l =* 1,2,*…*,*m* denotes the index for latent variables, *n* is the number of manifest variables and *m* denotes the number of latent variables, where $\:\:{\pi\:}_{kl}\left(\varvec{c}\right)$is the conditional probability that $\:{y}_{k}$, has a positive response given the values of the $\:m$ latent variables $\:{c}_{1},\ldots\:\ldots\:.,{c}_{m}$. ***c*** represents a vector of *m* latent variables that measure the different aspects of cost. In the measurement model, $\:{{\alpha\:}_{k0\left(s\right)}}^{\left(y\right)}\:and\:{\alpha\:}_{kl}$ represent intercepts of each category of observed items and factor loadings, respectively. The structural part is presented as

4 $$\:{c}_{l}={\theta\:}_{l1}\:cost+{\epsilon}_{l},\:l=\:\text{1,2}\ldots\:.m$$

where $\:{\theta\:}_{l1}\:$represents the factor loadings of the structural model.

The effect of different socioeconomic factors and other covariates on domestic violence are studied, by modelling violence as function of covariates within the full model as follows

5 $$\:violence=\:{\omega\:}_{v}\:Z+\xi\:,$$

where the vector $\:Z$ represents a set of covariates, such as: (education, salary, place of residence.,.etc.) and $\:{\omega\:}_{v}$ is a vector of regression coefficients

Finally, the effect of exposure to violence on cost is modelled within the full model as a function of domestic violence, as follows

6 $$\:Cost=\:\gamma\:\:Violence+\epsilon\:$$,

where $\:\:\gamma\:\:$is the regression parameter representing the effect of spousal violence on cost.

Measuring Domestic Violence and its cost, while estimating the effect of different socioeconomic covariates on violence is carried out simultaneously. The full model is fitted using Mplus software. Item Response Theory (IRT) approach is applied to fit the measurement part (latent trait model), while a structural equation model is implemented for the structural part. Maximum likelihood estimation is adopted for parameters estimation.

### Data and variables description

Data analysed in this study come from the Egypt Economic Cost of Gender-Based Violence Survey (ECGBVS), conducted in 2015 [24]. The aim of this survey was to study gender-based violence and its effects on reproductive and general health and wellbeing, as well as to assess the economic cost on the victim resulting from facing violence. It includes questions that cover different types of violence that women may face, in addition to addressing different aspects of cost resulting from such violence.

The ECGBVS used two questionnaires: a household questionnaire and an individual questionnaire for women aged 18–64. The household questionnaire included questions such as: age, sex, marital status and relationship to the household head. The household questionnaire covered all members in the household. These questions were used to identify women eligible for the individual questionnaire. The household questionnaire also gathered information about some housing characteristics (e.g. type of dwelling unit, the number of rooms, the material of floor, the source of water and the toilet facility) and on the ownership of consumer goods. These were used to create a wealth index to reflect the household’s socioeconomic level. The individual questionnaire included questions about characteristics of the selected women, working status, income, general health, reproductive health and different forms of violence, including violence perpetrated by husband/ family member.

The sample for the 2015 ECGBVS was designed to be representative of the Egyptian population. It included urban and rural areas from five regions: urban governorates, urban Lower Egypt, rural Lower Egypt, urban Upper Egypt and rural Upper Egypt, while frontier governorates were excluded from the sample since they represent less than 1% of the total population. The sample was designed to be a two-stage cluster sample. In the first stage, 1000 enumeration areas were selected from the sampling frame which depended on the 2006 population census. The areas were divided to be 45% for urban areas and 55% to rural areas. In the second stage, 22 households were selected from each of the urban areas and 21 from each of the rural areas. Accordingly, 21,448 households were selected for the survey and only 20,535 households were successfully interviewed. This yields a response rate of 97.3%. In these households, 20,157 women were identified as eligible for the individual interview. Out of these women, 20,000 were interviewed successfully, indicating a response rate of 99.2% [27].

Since this study focuses on spousal violence, it includes only the ever married or engaged women, who ever faced violence, leading to reduce the sample to 4,368 women. Questions related to most covariates were only addressed to currently married women. This data restriction resulted in reducing the sample to include 4,249 currently married women, thus allowing to study the effect of socioeconomic covariates on domestic violence and its consequent cost [28, p.14].

Variables are divided into three groups: manifest variables measuring domestic violence, manifest variables measuring cost, and finally covariates that do not contribute to measuring the main latent variables of interest but may externally affect them. A list of all variables can be found in the appendix, tables A1 – A4.

Domestic violence is measured across four dimensions: physical violence, sexual violence, psychological violence and economic violence. ECGBVS adopts a list of questions suggested by the WHO [29] to measure various dimensions of domestic violence. Also, the economic violence will be included in this study that is measured using four questions. In this paper, questions about facing different forms of violence in the previous 12 months are used, where all variables are binary [1: No, 2: Yes]. See appendix for the exact wording of questions used.

ECGBV survey included a set of questions that can help measure the cost that women may bear due to facing different types of violence. These questions were asked to all women who answered affirmatively to at least one of the previous violence questions (see appendix for questions wording). Approximately 23% reported that they have ever faced violence, while 77% have never faced any form of spousal violence. The selected variables include different aspects of cost such as monetary/ economic cost, cost on children who witness spousal violence, cost on health and cost on the woman’s wellbeing and her activities in general. The variables used in measuring cost are divided into two groups. The first group includes variables that were part of the survey, while the second group is derived variables from continuous variables in the data. Derived variables represent the amount of money spent due to the violence incident, such as: “Money spent on health services”, “Money spent on police services”, “Money spent on legal services” and “Accommodation cost”. After calculating those variables, they are categorized to have better fitting within the proposed modelling framework.

The variable “Money spent on health services” was created as a summation of three variables: “How much money was spent on health service?”, “How much money was spent on transportation for the health service?” and “How much money did you spend on treatment?”. The variable was then categorized into three categories. The variable “Money spent on police services.” was created based on a summation of two variables: “How much did you pay on transportation to police?” and “How much did you pay in the police station?”. Likewise, the variable “Money spent on legal services.” was created as a summation of three: “The cost of filing a lawsuit”, “Lawyer’s fees” and “Transportation cost to court”. Finally, “Accommodation Cost” represents the cost that a violated woman endures as a result of leaving her home and staying elsewhere. This variable is created by multiplying the cost of leaving home per day times the number of days she stayed out.

## Results

Before fitting the model, an exploratory factor analysis (EFA) was carried out [25, 30]. Exploratory factor analysis is used to extract different dimensions/ factors of domestic violence using 17 questions addressing exposure to different forms of violence during the 12 months preceding the survey. EFA is also used to determine which items are correlated the most and which items should be excluded from the analysis. The results of EFA show that the relationship between the observed items is best expressed using four factors labelled according to the type of violence that women face. The four types of violence are physical violence, psychological violence, sexual violence, and economic violence. Reliability and validity of the measurement model are also examined (see for example [31]). We use composite reliability (CR) (also called *construct reliability*) as a measure of internal consistency for our factors. Different references use different cutoff values for CR [32]. As a rule of thumb, a CR that is higher than 0.6 can be used as an indication of a reliable indicator. Table 1 shows that the Composite Reliability coefficient for all factors is higher than 0.6. Construct validity, calculated by the Average Variance Explained (AVE) is also reported in Table 1 An AVE value of 0.5 or higher is considered an indication of construct validity [33], giving more than 50% explanation for the variance. However, if AVE is less than 0.5, while CR is high, the construct can still be considered as valid, which is the case for some constructs reported in Table 1.

**Table 1:** Construct reliability and validity

Violence Constructs	CR	AVE
Economic Violence	0.884861	0.663133
Physical Violence	0.939610	0.724653
Psychological Violence	0.671870	0.377488
Sexual Violence	0.899992	0.750954
**Cost Constructs**	**CR**	**AVE**
Quality of Life	0.793917	0.388839
Children	0.943766	0.848483
Economic	0.869937	0.497599

**Fig. 2:**
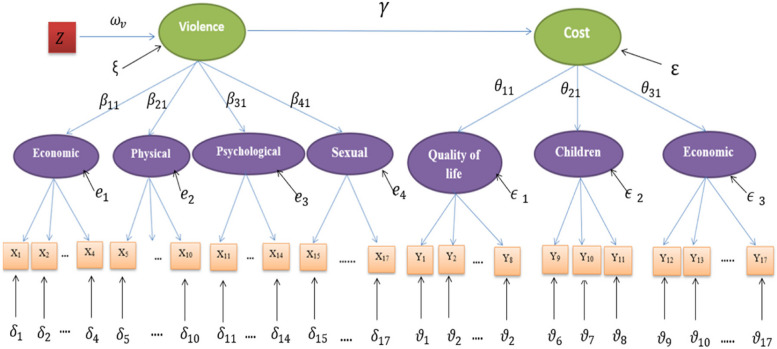
Path diagram of domestic violence and its consequent cost on violated women

Additionally, a confirmatory factor analysis (CFA) [25, 34, 35] was fitted for the cost of domestic violence, which included three factors, namely cost on women’s quality of life, cost on children, and economic cost. The relevance and reliability of the included cost items were first checked through running an exploratory factor analysis (EFA). Furthermore, the Composite Reliability is above 0.6 for all factors (see Table 1), and AVE either gives 50% or more explanation for the variance or is lower than 0.5 but associated with a high CR. Figure 2 presents the path diagram of domestic violence and its consequent cost based on EFA.

The model is measuring domestic violence against violated women and its consequent cost simultaneously, using data from 4,249 currently married women that have been subject to domestic violence; each is on two levels (measurement level and structural level) using a latent trait model fitted within the Item Response Theory approach. The two latent variables, Violence and Cost, are linked via a structural model. The first part of the model measures Domestic Violence against the violated currently married Egyptian women on two levels, as in the measurement level four types of violence capture the correlation between the different items. The structural level, “Violence” accounts for the correlation between the different types of violence. On the other hand, the consequent cost is measured onto two levels, in the measurement level three types of cost capture the correlation between the different items, similarly the structural level, “Cost” accounts for the correlation between the different types of cost. Then the relationship between violence and its consequent cost will be assessed as a structural equation model. Finally, the effects of different socioeconomic factors on domestic violence are assessed within the full structural model. Table 2 presents the fitted model for domestic violence and its cost.

**Table 2:** The Full Fitted Model

**Measurement Model for Domestic Violence**								
					${\mathrm a}_{\mathrm{iO}}$	*P*-value	${\mathrm a}_{\mathrm{ij}}$	*P*-value
** Economic**	X_1_	Preventing from work			2.599	0.000	1.080	0.000
	X_2_	Taking your money			6.329	0.000	3.147	0.000
	X_3_	Refusing to give money			3.634	0.000	2.495	0.000
	X_4_	Forcing to participate in home expenses			5.171	0.000	2.577	0.000
** Physical**	X_5_	Slapping or Throwing			-2.392	0.000	2.104	0.000
	X_6_	Pushing or Shoving			-1.426	0.000	2.851	0.000
	X_7_	Hitting with his fist			0.142	0.000	1.752	0.000
	X_8_	Kicking, Dragging or Beating			1.710	0.000	2.010	0.000
	X_9_	Choking or Burning			4.732	0.000	2.066	0.000
	X_10_	Threatening using a weapon			3.089	0.000	0.850	0.000
** Psychological**	X_11_	Insulting			-3.482	0.000	1.206	0.000
	X_12_	Belittling or Humiliating			-2.620	0.000	2.308	0.000
	X_13_	Doing things to scare you			0.397	0.000	0.266	0.000
	X_14_	Threatened to hurt you			2.163	0.000	0.961	0.000
** Sexual**	X_15_	Physical force to have sexual intercourse			1.574	0.000	3.908	0.000
	X_16_	Having sexual intercourse against your willing			1.493	0.000	3.069	0.000
	X_17_	Forcing to do something sexual that you found degrading or humiliating			3.581	0.000	1.972	0.000
**Structural Model for Domestic Violence**								
					${\mathrm\beta}_{\mathrm{jO}}$	*P*-value	${\mathrm\beta}_{\mathrm j1}$	*P*-value
** Violence**		Economic			-	-	0.677	0.000
		Physical			-	-	1.314	0.000
		Psychological			-	-	0.792	0.000
		Sexual			-	-	0.533	0.000
**Measurement Model for Cost**								
			${\mathrm a}_{\mathrm{iO}\left(1\right)}$	*P*-value	${\mathrm a}_{\mathrm{iO}\left(2\right)}$	*P*-value	${\mathrm a}_{\mathrm{ij}}$	*P*-value
** Quality of Life**	Y_1_	Having any injury	-0.385	0.000	-	-	0.524	0.000
	Y2	Taking time off from work	-2.122	0.000	4.593	0.000	0.113	0.003
	Y_3_	Stopping to do housework	2.374	0.000	-	-	1.627	0.000
	Y_4_	Husband taking time off work	2.708	0.000	4.822	0.000	0.133	0.007
	Y_5_	Husband stopping or reducing to offer domestic help	-1.731	0.000	5.552	0.000	0.167	0.000
	Y_6_	Going to police station	5.712	0.000	-	-	1.041	0.000
	Y_7_	Leaving your home	1.107	0.000	-	-	1.095	0.000
	Y_8_	Case filed in court due to incident	6.711	0.000	-	-	1.192	0.002
** Cost on Children**	Y_9_	Your children were absent from school	2.310	0.000	7.177	0.000	3.689	0.000
	Y_10_	Your children suffered from problems after incident	1.444	0.000	4.505	0.000	3.144	0.000
	Y_11_	Your children's educational performance affected	2.694	0.000	7.852	0.000	5.533	0.000
** Economic Cost**	Y_12_	Payments on Health Services	2.208	0.000	3.471	0.000	1.086	0.000
	Y_13_	Days taken off from work were paid	5.374	0.000	5.532	0.000	0.778	0.001
	Y_14_	Days your husband took off from work were paid	5.244	0.000	-	-	0.619	0.009
	Y_15_	Payments on renewing possessions	3.479	0.000	4.362	0.000	0.584	0.000
	Y_16_	Payments on Police Services	6.851	0.000	7.859	0.000	1.581	0.000
	Y_17_	Payments on Legal Services	7.711	0.000	8.593	0.000	1.700	0.000
	Y_18_	Payments on Accommodation	5.531	0.000	6.364	0.000	0.681	0.052
**Structural Model for Cost**								
			${\mathrm\beta}_{\mathrm{iO}\left(1\right)}$	*P*-value	${\mathrm\beta}_{\mathrm{iO}\left(2\right)}$	*P*-value	${\mathrm\beta}_{\mathrm{ij}}$	*P*-value
Cost		Quality of life	-	-	-	-	1.361	0.000
		Cost on Children	-	-	-	-	0.334	0.000
		Economic Cost	-	-	-	-	1.176	0.000
**Structural Model**								
					${\mathrm a}_{\mathrm{iO}}$	*P*-value	${\mathrm a}_{\mathrm{ij}}$	*P*-value
** Cost**		Violence			-	-	1.016	0.000

The fitted model in Table 2. shows that the economic violence has almost equally high loadings on X_2_, X_3_, and X_4_ which means that taking a wife’s money without her permission is highly participating in measuring economic violence against women, along with deprivation from money. Physical violence has the highest loadings on X_5_ and X_6_, indicating that pushing, shoving and slapping the wife have higher contributions in measuring physical violence than other items corresponding to more severe and less common forms of physical violence such as kicking, dragging, beating, choking, burning, or life threatening using a weapon. Psychological violence appears to be highly measured by humiliating wife (X_12_), compared to scaring (X_13_) and threatening (X_14_), which are again more severe and less common forms of violence against women. Sexual violence is highly loading on X_15_, which means that physically forcing a wife to have sexual intercourse against her will is the most contributing item to sexual violence. On the second (structural) level of the model, it is observed that physical violence contributes the most to measuring domestic violence against women, followed by psychological violence; while sexual and economic violence have lower contributions. This result highlights the ubiquity of the psychological and physical violence.

For consequent cost resulting from facing domestic violence, the model shows that the cost on woman’s quality of life is highly loading on Y_3_, followed by Y_7_ and Y_8_; indicating that stopping to do housework after exposure to any type of violence highly determines a woman’s quality of life, as well as leaving home and seeking legal support. The cost on children is highly loading on Y_11_ followed by Y_9_ and then Y_10_. This indicates that the affected educational performance of children after witnessing the incident is a major contributor to measuring the cost on children. By studying results of the fitted model, it is clear that economic cost is highly measured by cost of legal services (Y_17_), followed by cost of police services (Y_16_) and cost of health services (Y_12_).

On the second (structural) level of the model, it can be observed that economic cost contributes the most in measuring consequent cost of facing domestic violence, followed by cost on women’s Quality of Life; whereas cost on Children has the lowest contribution, which was unexpected. A very important finding of this model is that the cost of domestic violence is almost equally determined by the affected quality of life as by the economic burden resulting from violence. This highlights the importance of the non-monetary dimension of cost of domestic violence, which was discarded in most previous literature.

Cost of violence is allowed to depend on domestic violence via the structural model. This relationship is significant and positive, indicating that the higher the level of domestic violence that women face, the higher is the consequent cost that she bears, all forms of cost considered.

Finally, to study the effect of different socioeconomic factors on domestic violence of currently married violated Egyptian women, a structural equation model is fitted by regressing the latent factor “Violence” on the socioeconomic covariates within the whole fitted model. The model shows that the wealth index, women’s age at marriage, women’s working status and her salary have insignificant effect on domestic violence; that’s why they are excluded from the model. Table 3 shows the final fitted model for the effect of socioeconomic covariates on domestic violence.

**Table 3:** The Effect of different socioeconomic factors on domestic violence

Variable	Coefficient	S.E	*P*-value
Woman’s age	−0.009	0.004	0.028
Husband’s age	−0.011	0.004	0.002
Does your husband work?	−0.280	0.072	0.000
Husband’s Monthly Salary	0.070	0.025	0.005
Woman’s education_up to high school	−0.126	0.048	0.008
Woman’s education _above high school	−0.298	0.082	0.000
Husband’s education_up to high school	−0.114	0.047	0.016
Husband’s education _above high school	−0.291	0.073	0.000
Place of residence_urban	0.108	0.045	0.017
Living with husband’s parents or any of his relatives	0.226	0.042	0.000
Family members living nearby	−0.186	0.040	0.000

Table 3 shows that woman’s age and husband’s age almost equally negatively affect domestic violence, that is the older the wife and husband get, the less domestic violence a woman faces. A woman with an employed husband is less likely to be exposed to domestic violence than woman with a non-employed husband, holding other factors constant. There is a negative relationship between the level of education and domestic violence, that is the more the wife and/or husband are educated the less domestic violence endured. A positive relationship is noted between exposure to domestic violence and husband’s salary. The model also shows that urban women are more likely to face domestic violence than rural women, which is an unexpected result. However, this may be explained by the fact that violence here is multidimensional, and this association maybe due to a specific type of violence, such as economic violence, as concluded in previous studies, as in [14]. Additionally, the model shows that women who live with their husbands’ parents or other in-law suffer from higher exposure to domestic violence. Living close to women’s own family members, on the other hand, is associated with lower exposure to domestic violence.

To sum up, for socioeconomic covariates, the structural model shows that domestic violence is negatively affected by women’s and husband’s age, and by women’s and husband’s educational level, while it is positively affected by husband’s salary. Moreover, women living in urban areas and women who live with their husband’s parents or other in-laws are more likely to be exposed to domestic violence.

## Conclusion and discussion

Domestic violence against women, its determining factors, and its consequent costs are topics of interest to societies and researchers in various fields. This study focuses on measuring domestic violence and its consequent cost from different aspects, in addition to measuring the effect of different socioeconomic factors on domestic violence against Egyptian women. This study contributes to measuring violence and its consequent costs as multidimensional variables using Latent Trait Models. The main findings and implications of fitting this model are presented below.

Domestic violence is measured by summarizing four forms of violence: physical, psychological, sexual and economic violence, in a single continuous latent variable measuring “Domestic Violence”, that measures the scale of violence that Egyptian women face, not just whether or not they have ever faced violence, as in most previous studies. It is found that there is a positive relationship between the overall measure of Domestic Violence and all forms of violence, with psychological and physical violence having the strongest correlation with the overall measure.

Cost is measured by summarizing three forms of consequent cost of violence in another single continuous latent variable “Cost”, that measures the scale of cost that women bear as a result of being exposed to violence. The three dimensions of consequent cost of violence are economic cost, cost on children, and cost on women’s quality of life. These are measured as three continuous latent variables, via a number of observed questions. A positive relationship is detected between “Cost” and the three aspects of cost, but it can be highlighted that economic cost and cost on women’s quality of life have the highest contributions in measuring “Cost”.

The fitted SEM ensured the positive relationship between Cost and Domestic Violence. This means the higher the level of violence women face, the higher the level of the consequent costs, which can be reflected either economically, on quality of life, or on children.

In summary, for socioeconomic factors one can say that older couples, who are more educated, with an employed husband, living close to the woman’s family, are least likely to experience domestic violence against the woman. The risk of women facing domestic violence increases for younger couples, with less education, unemployment, and living with husband’s family. This is in line with results from previous studies, such as Alkan et al. [14–17] and Ribeiro et al. [18], who concluded that lower socioeconomic levels are associated with higher levels of violence.

It could be argued how the previous results highlight the importance of having deterrent laws to help reduce domestic violence and ensure that a violated woman can report violence freely and get her right. Nevertheless, the findings also show that psychological violence is no less prevalent or detrimental to women’s wellbeing than physical violence. This other pillar digs more into intrinsic social traditions and norms, including acceptance of violence against women, that may require different approaches and longer time to change the society’s perceptions. It can involve early awareness through educational systems, media campaigns, and even art productions such as movies and drama that address this issue,…etc. Both governmental and non-governmental organizations can play a major role in this part to mitigate these costs.

## Limitations and recommendations

As is the case for any research that involves modelling, developing the model in this study has faced a number of limitations. There were limitations related to the availability of the data. The most recent survey concerned with domestic violence in Egypt is the ECGBVS 2015 that was used in this study. No surveys have addressed this phenomenon extensively since then. The ECGBVS data contains missing values of both types, missing at random and missing not at random. This resulted in some reduction in the sample data used in the analysis.

Fitting a Latent Trait Model with ML estimator using Mplus software was time consuming, especially with the number of latent variables being higher than four. This required a high computational capacity for the numerical integrations. Moreover, convergence for the full model required several adaptations in the data. Chi square test for goodness of fit was invalid and thus not reported, due to the huge number of response patterns; causing a large fraction of expected frequencies to be less than 5.

In this study, non-violated women were excluded from the full-fledged simultaneous structural model, where focus has been shed on violated women and the resulting cost they endured. For future research, a model structure that compares characteristics of violated and non-violated women can be considered. A further contribution to this topic, is to fit a similar model using a Latent Class Model (LCM) instead of a Latent Trait Model (LTM), where Domestic Violence is constructed as a categorical latent variable. This can be specifically useful in studying the effects of socioeconomic covariates on the probability of being domestically violated. A comparative study is recommended to validate the results from fitting the proposed model to the ECGBVS dataset. The comparison may include other datasets from Egyptian national surveys, or further compare to societies with similar cultures and backgrounds, such as other countries in the MENA region or Turkey [14–17]. Comparison can extend to societies with different cultures, as for Western countries, in order to study differences and similarities of Domestic Violence, in various social environments. Structural latent models are promising tools that should be encouraged in other multidimensional social-science applications, such as in education and health, especially mental health.

## Supplementary Information

Supplementary Material 1.

## Data Availability

The Egyptian Central Agency for Public Mobilization and Statistics (CAPMAS) in cooperation with United Nations Population Fund (UNFPA), and the National Council for Women (NCW) conducted fieldwork and collected data for the ECGBVS, 2015, analysed in this study. The dataset [ECGBVS 2015] used in the analysis is available on the [CAPMAS portal] upon reasonable request at [https://censusinfo.capmas.gov.eg/Metadata-ar-v4.2/index.php/catalog/1336/study-description] CAPMAS reviews researchers’ request for the data, research objectives and proposals. Upon approval, data is shared with researchers at no cost.
