# Supplementary material for: Measuring domestic violence against Egyptian women and its consequent cost using a latent variable model
Source: BMC Womens Health. 2024 Dec 2;24:634. doi: 10.1186/s12905-024-03465-6 (PMC11613937; doi:10.1186/s12905-024-03465-6)
Supplement: Supplementary file 1 — Supplementary Material 1. [file 12905_2024_3465_MOESM1_ESM.docx]

**Appendix**

***Table A1. The Distribution of Manifest Variables to Measure Spousal Violence for Currently Married Women, Who Faced Spousal Violence in the 12 Months before Survey (2015 ECGBVS)***

| **Variable** | **Questions** | **Percentage of responding “Yes”** |
| --- | --- | --- |
| X_1_ | Does he prevent you from working (with the aim of earning money) against your will? | 6.4% |
| X_2_ | Does he take your money or income without your permission or against your will? | 1.9% |
| X_3_ | Does he refuse to give you money for home expenses, even if he has money to spend on other things such as cigarettes and alcohol? | 6% |
| X_4_ | Does he force you to participate in home expenses? | 2.3% |
| X_5_ | Has he slapped you or thrown something at you that could hurt you? | 44.2% |
| X_6_ | Has he pushed or shoved you? | 31.4% |
| X_7_ | Has he hit you with his fist or with something else that could hurt you? | 22.4% |
| X_8_ | Has he kicked you, dragged you or beaten you up? | 12.1% |
| X_9_ | Has he choked or burnt you on purpose? | 2.7% |
| X_10_ | Has he threatened to use or actually used a gun, knife or other weapon against you? | 3% |
| X_11_ | Has he insulted you or made you feel bad about yourself? | 85.3% |
| X_12_ | Has he belittled or humiliated you in front of other people? | 57.1% |
| X_13_ | Has he done things to scare or intimidate you on purpose? | 36% |
| X_14_ | Has he threatened to hurt you or someone you care about? | 8.1% |
| X_15_ | Has he physically forced you to have sexual intercourse when you didn’t want to? | 20.7% |
| X_16_ | Did you ever have sexual intercourse when you didn’t want because you were afraid of what he might do? | 20.3% |
| X_17_ | Has he forced you to do something sexual that you found degrading or humiliating? | 5% |
|  | **Number of Women** | **4294** |

***Table A2. The Distribution of Manifest Variables to Measure Cost Due to Facing Spousal Violence in the 12 Months Before Survey (2015 ECGBVS)***

| **Variable** | **Questions** | **Categories** | **Percentage** |
| --- | --- | --- | --- |
| Y_1_ | Have you had any injury after being exposed to violence? | No | 57.2 |
|  |  | Yes | 42.8 |
| Y_2_ | Did you to take time off work as a result of what happened? | No | 12.8 |
|  |  | Doesn't work | 86.3 |
|  |  | Yes | 0.9 |
| Y_3_ | Did you have to stop doing housework after this incident? | No | 88 |
|  |  | Yes | 12 |
| Y_4_ | Did your husband take time off work as a result of what happened? | No | 94.7 |
|  |  | NA (not working) | 4.6 |
|  |  | Yes | 0.8 |
| Y_5_ | Did your husband stop or reduce offering domestic help? | No | 17.3 |
|  |  | Doesn't help | 82.4 |
|  |  | Yes | 0.4 |
| Y_6_ | Did you go to the police station to report the incident? | No | 99.3 |
|  |  | Yes | 0.7 |
| Y_7_ | Did you leave your home after that incident? | No | 81.6 |
|  |  | Yes | 18.4 |
| Y_8_ | Is there any case filed in court due to reporting this incident? | No | 99.6 |
|  |  | Yes | 0.4 |
| Y_9_ | Have any of your children been forced to be absent from school as a result of this incident? | No | 76.2 |
|  |  | Not having children | 21.9 |
|  |  | Yes | 1.9 |
| Y_10_ | Did any of your children suffer from problems after this incident? | No | 70 |
|  |  | Not having children | 21.9 |
|  |  | Yes | 8.1 |
| Y_11_ | Did your children' educational performance affected after this incident? | No | 72.4 |
|  |  | Not having children | 21.9 |
|  |  | Yes | 5.7 |
| Y_12_ | Money spent on health services. | 0 | 89.6 |
|  |  | < 123 | 4.00 |
|  |  | >= 123 | 6.4 |
| Y_13_ | Were the days taken off from work as a result of what happened paid? | fully paid | 99.4 |
|  |  | partial paid | 0.1 |
|  |  | not paid | 0.5 |
| Y_14_ | Were the days your husband took off from work as a result of what happened paid? | fully paid | 99.5 |
|  |  | partial paid | 0 |
|  |  | not paid | 0.5 |
|  | How much did you pay on renewing your possessions? | 0 | 97.3 |
| Y_15_ |  | < 250 | 1.5 |
|  |  | >= 250 | 1.2 |
|  | Money spent on police services. | 0 | 99.4 |
| Y_16_ |  | <62 | 0.3 |
|  |  | >= 62 | 0.3 |
| Y_17_ | Money spent on judiciary services. | 0 | 99.7 |
|  |  | < 1120 | 0.1 |
|  |  | >= 1120 | 0.2 |
| Y_18_ | Accommodation Cost | 0  <350  >=350 | 99.6  0.2  0.2 |
|  | **Number of Women** |  | **4294** |

***Table A3. The Distribution of The Categorical Covariates (2015 ECGBVS).***

| **Variables** | **Categories** | **Percentage** |
| --- | --- | --- |
| Place of Residence | Urban | 37.4 % |
|  | Rural [R] | 62.6 % |
| Woman Education | Primary or less [R] | 46.4 % |
|  | Preparatory and Secondary | 40.4 % |
|  | Above Secondary | 13.2 % |
| Husband’s Education | Primary or less [R] | 38.2 % |
|  | Preparatory and Secondary | 44.1 % |
|  | Above Secondary | 17.7 % |
| Women Working Status | Working | 16.9 % |
|  | Not working [R] | 83.1 % |
| Does your husband work? | Yes | 90.2 % |
|  | No [R] | 9.7 % |
|  | Refuse to answer | 0.1 % |
| Wealth Index Quintiles | Lowest [R] | 20.2 % |
|  | Second | 21.2 % |
|  | Middle | 19.7 % |
|  | Fourth | 19.8 % |
|  | Highest | 19.0 % |
| Do any of your family members live nearby you? | Yes | 51.2 % |
|  | No [R] | 48.8 % |
| Do you live with your husband’s parents or any of his relatives? | Yes | 53.3 % |
|  | No [R] | 46.7 % |
| **Number of women** |  | **4249** |

[R]: reference category in regression model

***Table A4. Descriptive Statistics for Continuous Covariates (2015 ECGBVS).***

| **Variable** | **Minimum** | **Maximum** | **Mean** | **Median** | **St. Deviation** |
| --- | --- | --- | --- | --- | --- |
| Woman’ age | 18 | 64 | 36.20 | 35 | 9.781 |
| Woman’ age at Marriage | 11 | 48 | 19.75 | 19 | 3.994 |
| Husband’s age | 19 | 90 | 42.80 | 41 | 10.964 |
| Husband’s Monthly Salary | .00 | 10.00 | 1.4317 | 1.500 | 0.95998 |
| Woman’ Monthly Salary | .00 | 5.00 | 0.1671 | 1.200 | 0.47107 |
